# Assessment of household use of iodized salt and adequacy of salt iodization: a cross-sectional National Study in Saudi Arabia

**DOI:** 10.1186/s12937-018-0343-0

**Published:** 2018-02-28

**Authors:** Mushary H. Al-Dakheel, Hassan K. Haridi, Bushra M. Al-Bashir, Ali M. AL-Shangiti, Sulaiman N. Al-Shehri, Izzeldin Hussein

**Affiliations:** 1 Ministry of Health, Riyadh, Kingdom of Saudi Arabia; 2 Ministry of Education, Riyadh, Kingdom of Saudi Arabia; 3 Lipidomics and Nutrition Research Centre, London Metropolitan University, London, UK; 4 Iodine Global Network (IGN), Zürich, Switzerland

**Keywords:** Iodized salt, Household, Saudi Arabia

## Abstract

**Objectives:**

This study was conducted to assess household coverage with iodized salt in Saudi Arabia, and to determine adequacy of salt iodization.

**Methods:**

A school-based cross-sectional study using WHO 30-cluster survey methodology.

**Results:**

Analysis of 4242 salt samples using qualitative rapid test kit (RTK) revealed that 68.7% (95% CI 67.3–70.1%) were iodized with significant regional differences (*p* < 0.001). The highest iodized salt samples came from Makkah (82.3%), Riyadh (81.1%) and Maddinah (76.2%) regions, while the least iodized salt samples came from Hail (31.3%), Baha (53.0%), and Northern Borders (57.5%) regions. The national weighted proportion of households consuming iodizes salt was 69.8% (95% CI 69.4–71.2), which is below the Universal Salt Iodization (USI) goal (≥90% coverage). For validation, a quantitative iodometric titration method was used to analyze 775 representative salt samples screened iodized by RTK; iodine content of ≥15 ppm was found in 95.2% (95% CI 93.9–96.5) of samples with median iodine content 51 ppm (mean 50.4 ± 21.8). More than 70% of the iodized salt samples contained iodine concentration higher than the recommended national level (15–40 ppm).

**Conclusions:**

The study revealed inadequate consumption of iodized salt among Saudi households and explored marked regional heterogeneity. The majority of iodized salt samples contained iodine concentration more than the recommended level. These findings imply the need to launch a public awareness campaign on use of iodized salt. Legislation to ban production and sale of non-iodized salt sale for human consumption might be considered. A well-functioning monitoring system at factory level and surveillance system are crucially needed to ensure proper salt iodization and intake.

## Introduction

Iodine is essential for healthy brain development in the fetus and young child. Iodine deficiency negatively affects the health of women, as well as economic productivity and quality of life. It is the most common cause of preventable mental impairment throughout the world [1]. It has become very recognized that the consequences of iodine deficiency, including impaired brain development and low IQ in children, and nodules and hyperthyroidism in adults, can occur in the absence of clinical manifestation, such as cretinism and goiter [2]. Worldwide, it is estimated that one third (~ 1.88 billion) of the world population lives in areas where natural sources of iodine are low, with approximately 29.8% (241 million) school-age children globally are estimated to have insufficient intake of iodine [3]. There has been remarkable progress in the global effort to eliminate IDD. In 1993, WHO estimated that the prevalence of goiter due to iodine deficiency affected 110 countries globally. Over the past decade, the number of iodine-deficient countries has fallen from 54 to 25 and the number of countries with adequate iodine intake has increased from 67 to 116 [2].

Despite the scarcity of substantial historical data on iodine deficiency in Saudi Arabia, the two national surveys, carried out nearly two decades apart, have shown that Saudi population has a sufficient iodine nutrition at the national level, however, both surveys and other regional studies demonstrated a degree of mild to moderate iodine deficiency especially in the southern regions based in goiter prevalence indicator [4–7].

Food fortification is a public health policy aims to reduce the number of people with dietary deficiencies within a population. Salt is a good vehicle for iodization because it is consumed almost universally without seasonal variation; there are relatively few production facilities, simplifying quality control; technology for iodization is well established; consumer acceptability of iodized salt is high; and iodization is very inexpensive [8].

Universal Salt Iodization (USI) is a strategy recommended by the WHO and UNICEF Joint Committee on Health Policy since 1994 to ensure sufficient intake of iodine by all individuals. It indicate that all food-grade salt, used in household and food processing should be fortified with iodine as a safe and effective strategy for the prevention and control of iodine deficiency disorders in populations living in stable and emergency settings [1, 9]. This strategy has been implemented in more than 120 countries around the world, many of them have successfully eliminated iodine deficiency disorders or made substantial progress in their control [2, 10]. Saudi Arabia adopted this strategy as a recommendation of the first national survey about iodine deficiency disorders among Saudi population in 1994–1995 [4]. Salt iodization begins first at the level 70–100 ppm [11], then subsequently adjusted to a level of 15–40 ppm [12] responding to WHO recommendation [13].

Households coverage with iodized salt and monitoring quality of salt iodization are important key indicators in the implementations of USI programs [10]. Mapping disparities in household consumption of iodized salt and identifying areas with least coverage rates helps USI programs to target these areas. No data was available about household’s coverage with iodized salt and adequacy of salt iodization at the national level in Saudi Arabia; these were not a part of data collection in the 1994–1995 national survey [4]. The present study aimed to estimate the proportion of households consuming iodized salt in Saudi Arabia at the national and regional levels and to assess the adequacy of salt iodization.

## Methods

This study was based on the dataset of the National Survey on Iodine Deficiency Disorders conducted in 2012, which provided valuable information on the situation of iodine nutrition status in Saudi Arabia [5]. The survey was a cross-sectional school-based study adopted the ‘30 cluster’ sampling methodology recommended by WHO/UNICEF/ICCIDD [14].

### Setting

The Kingdom of Saudi Arabia (KSA) is widespread country constituting the bulk of the Arabian Peninsula with a land area of nearly two million km^2^. The land is primarily desert with rugged mountains in the southwest. The country is broadly divided into five geographical-areas, represented by the Central, Eastern, Western, Northern and Southern provinces with thirteen administrative regions. Central province includes, Riyadh and Qaseem regions; Western province includes, Makkah and Maddinah regions; Eastern province includes Eastern region; Northern province includes, Hail, Jouf, Northern Borders and Tabouk regions and Southern province includes, Aseer, Jizan, Baha and Najran regions. The population estimation according to 2010 census was ~ 27 million [15].

### Study population and sampling

Nearly a 100% primary school enrollment for both sexes in Saudi Arabia [16], making the school setting appropriate for ascertaining population-representative samples of children.

School children aged 8–10 years was the target population of this survey. Sampling frame of the study included all primary schools of the country (public and private) with a total of 13,626 schools, representing the primary sampling units (PSUs) [16].

Three stage random sampling was employed. In the first stage; the country was stratified into the five basic geographical areas; the Central, Eastern, Western, Northern and Southern provinces in order to carry out a separate subnational survey for each geographical area. In the second stage, a probability proportionate to size (PPS) sampling method was employed; thirty clusters were drawn randomly from the list of the PSUs in each geographical area. Every cluster represented a pair of schools; a male school and its neighbor female one, since schools in Saudi Arabia are unisex. In the third stage, thirty children for every cluster were chosen at random by systematic random technique, of whom, fifteen males and fifteen females children. Sample size for every geographical area survey was calculated at (*n* = 769) on the ground of an absolute precision of ±5% with 95% confidence interval and design effect of 2 for anticipated 50% iodized salt use among households (to maximize sample size, since we expected large regional disparities). Sample size was increased by 10% more to compensate for non-response.

A total of 30 clusters for every geographical area was completed, with 932, 968, 683, 1029 and 617 salt samples were collected from central, western, eastern, northern and southern provinces respectively (Table 1).

**Table 1:** Sample distribution and results of 4242 salt samples screened for iodine content by rapid test kit (RTK) classified by the five geographical provinces and the 13 administrative regions in Saudi Arabia, 2012

Locality	No. of Clusters	No. of Salt Samples	% Iodized salt by RTK (95% CI)
Regions in Central Province			
- Riyadh	24	718	81.1 (78.0-83.8)
- Qaseem	6	214	75.2 (68.9-80.9)
Total	30	932	79.7 (77.0-82.2)
Regions in Western Province			
- Makkah	22	691	82.3 (79.2-85.1)
- Maddinah	8	277	76.2 (70.7-81.1)
Total	30	968	80.6 (77.9-83.0)
Regions in Eastern Province			
Total	30	683	63.8 (60.1-67.4)
Regions in Northern Province			
- Hail	8	294	31.3 (26.0-36.9)
- Northern Borders	5	186	57.5 (50.1-64.7)
- Tabouk	11	354	66.7 (61.5-71.6)
- Jouf	6	208	60.6 (53.6-67.3)
Total	30	1042	53.8 (50.8-56.9)
Regions in Southern Province			
- Jizan	11	224	66.5 (59.9-72.7)
- Asseer	13	173	61.8 (54.2-69.1)
- Baha	3	117	53.0 (43.5-62.3)
- Najran	3	103	74.8 (65.2-82.8)
Total	30	617	64.0 (60.1-67.8)
National	150	4242	68.7 (67.3-70.1)
			69.8 (69.4-71.2)^a^

Abbreviations: *RTK* rapid test kit; *CI* confidence interval

^a^Adjusted national estimate to account for regional population weights

There was a significant difference in iodized salt samples among geographical provinces (χ^2^ = 237.1; df = 4; *P* < 0.001)

### Data collection

Data on participant age, gender, educational class, locality were collected. Salt samples of about 20 g of routinely consumed salt, were collected by requesting all participants to bring from their homes. A clean air tight plastic pouch was provided to each enrolled child for this, so that there was no loss of iodine during sample transport. Each salt sample was screened qualitatively for iodine content using a rapid salt testing kits (RTK) [17]. These consist of a stabilized starch-based solution. One drop of the solution dripped on a teaspoon of salt containing iodine produces a blue/purple color change. Coloration indicates that iodine is present > 15 ppm with inability to accurately identify salt iodized at < 15 mg/kg of iodine. In addition, the rapid test kits cannot identify overiodized salt at concentrations that may exceed 80–100 mg/kg [3]. Salt samples were classified qualitatively as iodized or non-iodized. From the samples identified as iodized by the RTK, 25% were randomly selected for iodine level determination using the quantitative iodometric titration method [17]. One person analyzed all the samples in duplicate. Adequately iodized salt was operationally defined as the salt containing 15 ppm of iodine or more for reporting RTK results, however, a well-functioning iodization is indicated when iodine concentration in salt of 15–40 ppm was met by iodometric titration method [17]. All reagents and salt analysis were done centrally under quality control measures in the Central Laboratory for Nutrition in Riyadh.

### Statistical analysis

Data processing and statistical analyses were performed using the Epi Info statistical package version 7 (CDC, Atlanta, GA, USA). General tabulation including frequency distribution were used with 95% confidence interval (CI). Salt iodine concentration and other continuous variables were expressed in median with interquartile range (IQR) and mean (± SD). Chi-square test was used to test for differences in proportions. National estimates of coverage were weighted according to the total number of children per region compared to the national total. Analysis for complex sampling design took into consideration sampling stratification, clustering and regional weights and permitting estimates adjustment. The mean iodine concentration measured by iodometric titration for salt subsample was calculated in simple arithmetic for regional and national estimates. All tests were two-sided and the level of significance was set at *p* < 0.05.

### Ethical considerations

The ethical issues of this study was reviewed and approved by the Ethical Committee of the Ministry of Health, KSA. Approval of Education Authority was obtained. A written informed consent was obtained from the parents/guardians of participating children.

## Results

Out of the total of 4242 salt samples collected, 68.7% (95% CI 67.3–70.1%) were found iodized using rapid test kit (RTK) (Table 1), with significant provincial differences (*p* < 0.001). The northern province had the lowest percent (53.8%) of iodized salt samples, whereas western province was the highest (80.6%). At the level of administrative regions; significant differences (*p* < 0.001) were observed in iodized household salt use, varying from highest 82.3% in Makkah region, 81.1% in Riyadh region, 76.2% in Maddinah region, together have more than half of population weight of the country to least proportions of households consuming iodized salt as 31.3%, 53.0% and 57.5% for Hail, Baha and Northern Borders regions respectively (Table 1), all of them have a limited population weights.

Iodometric titration method was used for validation of iodine content in samples tested by the RTK. A representative sample (*n* = 775) from the total samples screened positive for iodine by RTK were tested. The results revealed adequate (≥ 15 ppm) iodine content of salt in “95.2%” (95% CI 93.9–96.5) of the samples, with a mean ± SD 50.4 ± 21.8 and median 51 ppm concentration (Table 2). There was a significant difference in iodine content of iodized salt samples among different geographic provinces (*P* < 0.001). The proportion of households consuming adequately iodized salt was estimated at: 75.9% (95% CI 73.2–78.7), 76.7% (95% CI 74.0–79.4), 60.7% (95% CI 57.0–64.4), 51.2% (9% CI 48.2–54.2), and 60.9% (95% CI 57.1–64.8) for Central, Western, Eastern, Northern and Southern provinces respectively, with weighted national estimate at 69.8% (95% CI 68.4–71.2). An important finding in Table 2 shows that more than two third (70.7%) of the overall iodized salt samples analyzed by the quantitative iodometric titration method exceeded the upper level of the national recommended level of iodine salt concentration (15–40 ppm) iodine. Percentage of salt samples over-iodized with iodine content > 40 ppm were 55.2%, 84.1%, 66.9%, 73.1%, and 76.0% for Central, Western, Eastern, Northern and Southern provinces respectively.

**Table 2:** Iodine content of salt tested by iodometric titration method in a representative salt sample (n= 775) positive for iodine content by rapid test kit (RTK) at different geographical provinces and national level in Saudi Arabia, 2012

Geographical areas	No. of Salt Samples	% of salt samples at various iodine concentrations (ppm)			Mean (SD)	Median (IQR)
		<15	15-40	>40		
Central province	172	7.0	37.8	55.2	42.76 (21.30)	43.5 (27.0-54.0)
Western province	182	1.6	14.3	84.1	54.32 (16.88)	53.0 (44.0-65.0)
Eastern province	142	12.7	20.4	66.9	51.33 (27.91)	54.0 (29.0-69.0)
Northern province	154	1.3	25.6	73.1	52.10 (21.05)	52.0 (38.0-64.0)
Southern province	125	0.0	24.0	76.0	52.44 (18.98)	51.0 (41.0-64.0)
National	775	4.8	24.5	70.7	50.40 (21.80)	51.0 (37.0-63.0)

Abbreviations: *RTK* rapid test kit; *SD* Standard Deviation; *IQR* Interquartile Range 25%-75%

There was a significant difference in iodized salt samples among regions (χ^2^ = 68.42; df = 8; *P* < 0.001).

## Discussion

In an effort to elucidate the coverage and quality of iodized salt use among households in Saudi Arabia, we conducted the present national cross-sectional survey. Our results underscored three significant findings with implication to the national USI program: (1) a national coverage below the USI target; (2) coverage varies considerably between regions; and (3) improper salt iodization.

To our knowledge, this study is the first to report national representative data on household coverage with adequately iodized salt in Saudi Arabia. Before this survey a scarce data on the households’ coverage with iodized salt were only identified, coming from few regional studies and revealed a wide range of coverage. A report from Hail at the north of Saudi Arabia (2004); described the pattern of household salt consumption and pointed to the prevalent pattern of consumption of the non-iodized salt in that area. Only 27.7% of households were consuming iodized salt as an exclusive source of salt, 31.6% were consuming non iodized salt, while 40.7% were consuming iodized salt as a table salt and not for cooking purposes (Haridi HK et al.: Project Early Detection & Health Education of Thyroid Cancer in Hail, 2004, unpublished). On the other hand, at the southern province, a study in Jizan region, 2010, reported almost all households were consuming iodized salt [6]. A recent study from Aseer region at southern province, reported 77.5% coverage rate [7].

The first significant findings in our study is that: despite the relatively reasonable coverage at the national level where 69.8% of households were consuming iodized salt, yet, it is still far from the criterion 90% coverage of USI goal by WHO [17]. This coverage is comparable to the neighbor Oman (68.5%), higher than Sudan (9.5%), Morocco (21.2) and Yemen (29.5%), but lower than Egypt (77.7%), Lebanon (74.8%) and Tunisia (96.7%) [18].

National production of iodized salt in Saudi Arabia is high exceeds the domestic demand and exported to the international market. Salt production in Saudi Arabia was about two million metric tons in 2012 according to British Geological survey and ranked 23 in the leading salt producers worldwide [19]. As an open market, the imported iodized salt in Saudi Arabia is also available. Both local and imported iodized salt have an affordable price.

Regulations in Saudi Arabia are not banning the production and distribution of non-iodized salt. Availability and easy accessibility to coarse non iodized salt, with low price and big quantity packages competes the exclusive availability of the iodized salt and impedes the achievement of the recommended USI goal. The coarse salt available in the market is produced locally from desert salt lakes or rocky salt. It is very deficient or have no iodine and traditionally consumed by households for many decades and considering it as natural and tasty. This imply that public awareness campaigns about the importance of iodized salt is especially needed in areas where non iodized salt use is common. Legislation to ban the non-iodized salt sale may be of value and proved effective in other countries [20].

The second significant findings in our study is the wide regional disparities in iodized salt use among households, with none of the 13 regions in the country attained WHO/UNICEF/ICCIDD USI criterion of “≥90% of households consuming adequately iodized salt” [17]. Inequalities in coverage ranged between 31.3% in Hail region at the Northern province to 82.3% in Makkah region at Western province. This marked regional variation imply the need to study factors affecting such disparities in terms access, demand and preferences of households as regard salt consumption. Worth noting that most regions with low iodized salt use were low density population regions and the highest coverage was in the central and western regions, the highly populated.

The third significant findings in our study is the excess amount of iodine in iodized salt that exceeds the range of the recommended fortification (15–40 ppm). The iodine content of iodized salt measured by iodometric titration method, revealed 51 ppm median (mean 50.4 ± 21.8), with more than two third (70.7%) of iodized salt samples contained more than 40 ppm iodine which may leads to iodine over intake with a risk of adverse health consequences like iodine-induced hyperthyroidism and autoimmune thyroid diseases [10, 13, 17, 21]. This draws the attention to the need for a good monitoring system to ensure commitment and proper quality control systems of salt producers. Several salt producers, still marketing salt with iodine according to the previous code (70–100 ppm). It is essential to change legislation to mandate the iodine salt adjustment (15–40 ppm) instead of just recommending it.

Lessons from international experience indicated that policies, laws, and agreements requiring all edible salt to be iodized, effective inspection and enforcement systems, and political advocacy and scientific support from community leaders are critical to any national iodine deficiency disorders elimination program requiring salt iodization [2]. Helping small-scale producers of the non-iodized coarse salt in qualifying the process of production and fortifying their product by the recommended amount of iodine, may be helpful in rapid achieving the goal of the iodized salt full coverage.

Despite the known benefits of appropriate consumption of iodine, some concern exists that widespread salt iodization could potentially lead to excess iodine intake [21]. Monitoring of food-grade salt quality is essential to ensure both efficacy and safety of the process of iodine fortification [10]. On the other hand, as populations reduce salt intake to reduce the risk of elevated blood pressure, hypertension and stroke, there may be increase in iodine deficiency disorders [22, 23]. It is crucial to recognize that salt reduction and salt iodization strategies are not contradictory [24]. As salt consumption is reduced, iodine levels should be increased to ensure populations continue to consume enough iodine. Meanwhile, the objective of salt iodization programs is not to encourage increased consumption of salt but to ensure it is all iodized [24].

The strength of the present study is being conducted in five separate subnational provincial surveys that allowed for better regional mapping of the households’ coverage with iodized salt. However, our study might have had some limitations: First, we experienced some difficulty to recruit the ideal number of children in some clusters in Jizan at the southern province because of high absenteeism due to heavy rain during the survey time. Yet, the small number of clusters and limited population weight of the locality does not compromise our general interpretation of the results. Second, the study did not explore factors affecting household consumption of iodized salt, which will be addressed in a subsequent household survey, focusing on regions with least coverage. We acknowledge that the present study is somewhat dated and may not reflect the current situation with regard to salt iodization in Saudi Arabia. However, the information contributes to the process of periodic assessment of the progress towards USI efforts in the country.

## Conclusion

The study highlighted inadequate consumption of iodized salt among Saudi households and explored regional heterogeneity. The majority of iodized salt samples contained iodine concentration more than the recommended level. These results, suggests the need to ban non-iodized salt sale and to launch community awareness campaigns especially in areas where non-iodized salt use is common. Establishing a proper monitoring system at factory level and surveillance system are crucially needed to ensure proper salt iodization and intake.
